# Perception and Evaluation of 23 Positive Emotions in Hong Kong and the Netherlands

**DOI:** 10.3389/fpsyg.2021.579474

**Published:** 2021-05-28

**Authors:** Rui Sun, Wai Kai Hou, Bryant P. H. Hui, Nicolson Yat-Fan Siu, Tiarah Engels, Disa A. Sauter

**Affiliations:** ^1^Department of Psychology, University of Amsterdam, Amsterdam, Netherlands; ^2^Department of Psychology, The Education University of Hong Kong, Tai Po, Hong Kong; ^3^Department of Sociology, The University of Hong Kong, Pok Fu Lam, Hong Kong; ^4^Department of Counselling and Psychology, Hong Kong Shue Yan University, North Point, Hong Kong

**Keywords:** positive emotions, culture, individual perception, societal evaluations, positive psychology, emotions

## Abstract

Positive emotions are linked to numerous benefits, but not everyone appreciates the same kinds of positive emotional experiences. We examine how distinct positive emotions are perceived and whether individuals’ perceptions are linked to how societies evaluate those emotions. Participants from Hong Kong and Netherlands rated 23 positive emotions based on their individual perceptions (positivity, arousal, and socially engaging) and societal evaluations (appropriate, valued, and approved of). We found that (1) there were cultural differences in judgments about all six aspects of positive emotions; (2) positivity, arousal, and social engagement predicted emotions being positively regarded at the societal level in both cultures; and (3) that positivity mattered more for the Dutch participants, although arousal and social engagement mattered more in Hong Kong for societal evaluations. These findings provide a granular map of the perception and evaluation of distinct positive emotions in two cultures and highlight the role of cultures in the understanding how positive emotions are perceived and evaluated.

## Introduction

Most people report being quite happy most of the time (Diener and Diener, 1996), and positive emotional experiences are important because of they play a key role in well-being and health (Bastian et al., 2014). However, not everyone appreciates the same kinds of positive emotional experiences. Besides, little is known about how people perceive and evaluate specific positive emotions such as contentment, relief, pride, and gratitude.

Generally speaking, emotions can be analyzed on at least four levels: individual, dyads, group, and culture (Keltner and Haidt, 1999). In the current work, we focus on the *perception* of emotions at the individual level in terms of positivity, arousal, and social engagement as well as the *evaluation* of emotions at the societal level in terms of whether they are valued, approved of, and regarded as appropriate; we also tested how judgments across the two levels relate to one another. Specifically, the present study examined the perceptions and evaluations of 23 different positive emotions across two cultural contexts: Hong Kong and Netherlands.

## Individuals’ Subjective Perceptions of Emotions Across Cultures

Culture is a socially transmitted constellation of practices, symbols, and values that shape how people think, feel, and behave (Markus and Conner, 2013). A major distinction exists between individualistic and collectivistic cultures. In individualistic cultures, such as North America and Western Europe, ideas relating to independence, including personal achievement and goal pursuit, tend to be emphasized. In contrast, in collectivistic cultures, such as in East Asia, ideas emphasizing social harmony and interdependence are highly regarded (Markus and Kitayama, 1991). These differences in cultural values affect how individuals perceive emotions in terms of various attributes, such as positivity, arousal, and social engagement.

Positivity is the intrinsic attractiveness or pleasure associated with an event, object, or situation (Frijda, 1986). People generally prefer positive emotions over negative ones regardless of cultural background (Larsen, 2000). However, positive emotions are more desirable in Western cultures than in East Asian cultures (Eid and Diener, 2001), and how positive emotions are seen also depends on culture. For example, when asked to describe features of positive emotions, Americans mainly focus on the positive aspects, and Japanese people are more likely to mention negative aspects as well (Uchida and Kitayama, 2009). Thus, although most people from both Western and East Asian societies *like* positive emotions, they may see positivity from different angles. To date, only a handful of specific positive emotions have been examined in terms of positivity perception (Farmer and Kashdan, 2012; Miyamoto et al., 2014). Hence, little is known about the wider range of positive emotions that are increasingly being examined in the research literature. In the current study, we sought to test and compare perceptions of positivity of 23 different positive emotions across two cultures.

Arousal corresponds to the feeling that environmental demands require energy and mobilization (high arousal) or allow rest and recuperation (low arousal; Russell, 2003). Some cross-cultural consistency has been found in perceptions of arousal of positive emotions. For example, “excited” is rated as high arousal and “serene” and “content” judged as low arousal across Estonian, Greek, and Polish participants (Russell et al., 1989). Moreover, excitement, enthusiasm, and elation are frequently considered high-arousal positive emotions and calm, peacefulness, and serenity low-arousal positive emotions across cultural groups (Tsai, 2007). However, similar to research on perceptions of positivity, there is a lack of examination across cultures of the perception of arousal levels for a wide range of distinct positive emotions. In the current study, we, therefore, also measured perceptions of arousal by Western and East Asian participants of a wide range of positive emotions.

Culture also affects social relationships. In East Asian cultures, social themes related to social harmony are more salient, whereas autonomy and personal achievements and goals are central in Western societies (Markus and Kitayama, 1991). Emotions centered around social interdependence and relational harmony have been termed *socially engaging* (e.g., sympathy and respect), and emotions that are grounded in independence and autonomy are considered *socially disengaging* (e.g., pride; Kitayama et al., 2006). In East Asian cultures, people report experiencing socially engaging positive emotions more frequently and intensely than socially disengaging emotions. In Western cultures, socially disengaging positive emotions are experienced more intensely and more commonly than socially engaging emotions (Kitayama et al., 2000, 2006). Moreover, experiences of positive, socially engaging emotions are related to better well-being in East Asia, and experiences of positive, socially disengaging emotions are related to better well-being in Western societies (Kitayama et al., 2000). However, little research to date has directly examined which positive emotions are perceived as socially engaging versus disengaging across cultures. The present study sought to contribute to filling this gap.

## Societal Evaluation of Emotions

By shaping daily interactions and goals, culture influences not only how emotions are perceived by individuals, but also how they are evaluated in societies. For instance, anger promotes autonomy and independence and is, thus, normative in individualistic cultures, whereas it is not desired in collectivistic cultures because it violates social harmony (Boiger et al., 2013). Besides this, how emotions are evaluated in one’s culture can shape the emotional experiences one reports; when people think that their emotions are viewed positively by others, they report experiencing those emotions more readily (Evers et al., 2005). However, several questions remain unanswered that the present study sought to address. First, we sought to establish the elements based on which people evaluate positive emotions and whether that differs across cultural groups. Second, we tested whether the level of perceived positivity, arousal, and social engagement would map onto how positive emotions are evaluated in society. Third, we examined whether, given that culture likely influences the perceptions of positive emotions, there would be cultural differences in perception–evaluation links.

Previous research has examined associations between arousal levels of positive emotions and culturally specific evaluations of those emotions. Tsai et al. (2006) show that high-arousal emotions were valued more among European Americans than Asian Americans, and low-arousal emotions were valued more among Asian Americans than European Americans. Building on that work, the present study asked participants from different cultural backgrounds to judge their society’s evaluations of those emotions in terms of appropriateness, being valued, and approved of.

## Current Study and Hypotheses

The current study focuses on the cultural influence on individuals’ perception and societal evaluations of positive emotions as well as the links between perceptions and evaluations. We first examined the extent to which Easterners and Westerners would *perceive* positive emotions similarly vs. differently on positivity, arousal, and social engagement and whether East Asian society and Western societies would *evaluate* positive emotions similarly or differently on appropriateness, being valued, and approved of. Second, we tested the links between individual perceptions and societal evaluations of positive emotions in each cultural group, attempting to establish whether different kinds of individual-level perceptions would contribute to evaluations of positive emotions at the group level. We also sought to probe cultural differences in the perception–evaluation links.

Specifically, we asked students from Hong Kong and Netherlands, representing Eastern and Western cultures, respectively, to rate 23 positive emotions on positivity, arousal, and degree of social engagement; they were also asked to report how they thought their culture saw each emotion in terms of the degree to which it was considered appropriate, valued, and approved of. In the present work, participants’ country^1^ of residence is employed as a measure of culture as it has been proven to be a robust measure to account for cultural differences (Crotts and Litvin, 2003).

Based on the existing literature, we formulated three hypotheses:

*H1*: Emotional arousal is perceived consistently across cultures, whereas positivity and social engagement as well as societal evaluations are rated differently by Westerners and East Asians.

*H2*: Individual perceptions (positivity, arousal, and social engagement) are positively related to the evaluation (appropriateness, valued, and approved of) of positive emotions in both Westerners and East Asians.

*H3*: The perception–evaluation relationship differs across the two cultural groups. Specifically, for Westerners, the positivity (3a) and arousal (3b) of positive emotions are more strongly linked to perceived societal evaluations, and for East Asians, perceptions of social engagement are more strongly linked to societal evaluations (3c).

## Methods

### Participants

An *a priori* power calculation for repeated measures, between-factors MANOVA showed that a total of 386 participants was needed to obtain power of 0.95, α error probability = 0.00036 (Bonferroni corrected), correlation among repeated measures = 0.1, at an expected small effect size 0.1. A total of 244 students from Hong Kong (*n*_m__an_ = 113, *n*_woman_ = 122, Prefer not to say = 9) and 253 students from Netherlands (*n*_ma__n_ = 122, *n*_woman_ = 129, Prefer not to say = 2) took part. There was no difference in gender distribution across groups, *Chi-square* = 4.83, *p* = 0.09. Hong Kong Chinese participants (*Mean*_age_ = 20.46, *SD*_age_ = 2.11) on average were 1 year younger than Dutch participants (*Mean*_age_ = 21.45, *SD*_age_ = 3.59), *t*(424) = −3.93, *p* < 0.001.

### Materials

Based on research on positive emotions, 23 positive emotions were included: admiration, amusement, awe, determination, euphoria, excitement, gratitude, hope, inspiration, interest, moved, surprise, schadenfreude, pride, relief, respect, pleasure, tenderness, triumph, contentment, compassion, peace, and connected (e.g., Weidman et al., 2017; Weidman and Tracy, 2020). The selection of emotions was based on two criteria: (1) that people generally feel positive when experiencing the emotion, and (2) that the affective state is relatively brief (typically seconds or minutes rather than hours or days). To ensure that participants would understand the meaning of each emotion term, participants were provided with the definition of each target positive emotion term before they made judgments. Please see Supplementary Table 1 for the terms and definitions in English, Dutch, and Cantonese.

### Procedure

The protocol received ethical approval from the Ethics Committee of the Department of Psychology at the University of Amsterdam, and all participants provided digital consent. The study was conducted online in participants’ mother tongue and lasted approximately 15 min. All materials were translated from English to Cantonese and Dutch and back-translated to English (Brislin, 1970).

Participants rated the 23 emotions in a random order. For clarity, operational definitions of emotional arousal (“Emotion arousal is a subjective state of feeling activated or deactivated. In a high-arousal emotional state, one may have a quicker heartbeat and sweaty palms; in a low-arousal emotional state, the body feels calm”) and socially engaging/disengaging (“Socially engaging emotions help with harmony in our relationships with those around us, while socially disengaging emotions separate us from others in a relationship”) were provided.

For each emotion, participants answered six questions on nine-point Likert scales. Three of the six questions concerned their *own* perception: ‘‘How positive do you think this emotion is?’’ ‘‘How aroused do you think this emotion is?’’ and ‘‘How socially engaging do you think this emotion is?’’ The other three questions probed how participants believed their *society* sees each emotion: ‘‘How appropriate is this emotion seen as being in your society?’’ ‘‘How valued is this emotion in your society?’’ and ‘‘Do people in your society approve of this emotion?’’ For five out of the six questions, options ranged from 1 (not at all) to 9 (very much). For the question on social engagement/disengagement, options ranged from 1 (very disengaging) to 9 (very engaging)^2^.

## Data Analysis and Results

### Data Preparation

We used R studio 3.1 for data analysis. We first sought to control for potential differences in the ways that the members of the two cultural groups use rating scales and, therefore, conducted within-culture standardization for the two groups separately (Fischer, 2004). For each culture, we first subtracted the grand mean (the mean across all items and all individuals in the group) from each response and then divided it by the grand standard deviation (the standard deviation across all items and individuals in that group). Participants’ judgments for each of the 23 positive emotions are listed in Supplementary Tables 2, 3 (raw scores) and (corrected for cultural response biases). Schadenfreude (the feeling of enjoying something bad happening to another person) was not rated as a positive emotion even though it fit our definition: 2.89 (Hong Kong Chinese) and 3.18 (Dutch) on a nine-point scale with five being the midpoint and nine being maximally positive. Therefore, it was excluded from further data analysis; the ratings of schadenfreude were consequently not used for calculating the grand mean and grand standard deviation.

### Descriptive Results of Ratings by Question Type

Prior to the hypothesis tests, we report the descriptive results of the six questions for Hong Kong and Netherlands. For these analyses, we averaged the ratings across all 22 emotions per group. We calculated Cronbach’s alpha for each of the six questions for Hong Kong and Netherlands, separately. All alpha values were above 0.86, showing good reliability (see Supplementary Table 4A). The average ratings for each question type in each country are reported in Supplementary Table 4B.

#### Hypothesis Testing

*H1:* We first tested the H1 that there would be cultural consistency for arousal ratings and cultural differences for the other five types of ratings on the emotion ratings. We then ran multivariate regression models six times in each model, using the country as predictor and ratings for all positive emotions as outcomes (e.g., the arousal ratings for all 22 emotions). For each model, we first tested the multivariate effect for the 22 emotions as a group in relation to country (Supplementary Table 5A); we then tested the country-emotion rating relationship for each emotion. Because there were 22 emotions as outcome variables, in each model, the *p*-value was corrected for multiple comparisons using the Bonferroni-Holm method (reported in Supplementary Table 5B).

The multivariate results show that, for all six ratings, including the arousal ratings, there were clear cultural differences. Contrary to our hypothesis that Hong Kong Chinese and Dutch participants would rate emotions similarly for *arousal*, we found that positive emotions were rated as higher in arousal in Hong Kong than in Netherlands. In terms of specific emotions, awe, gratitude, moved, tender, and triumph were statistically rated more aroused by Hong Kong participants than Dutch participants. The other emotions were rated similarly across the two countries.

Hong Kong Chinese participants’ ratings on positivity and social engagement were higher than those of Dutch participants. For *positivity*, it is worth noting that, even though statistically overall these emotions’ positivity was rated higher in Hong Kong (0.204) than in Netherlands (0.195), there was a large discrepancy in which emotions were rated as more positive in the two countries. Awe, hope, moved, and tender were rated higher in Hong Kong than in Netherlands, and amusement, inspiration, interested, positive surprised, relief, and compassion were rated higher in Netherlands than in Hong Kong. Similarly, for *social engagement*, overall ratings were higher in Hong Kong (−0.023) than in Netherlands (−0.075), but there were large discrepancies across emotions. Amusement, compassion, and connected were rated more as socially engaging in Netherlands than in Hong Kong, and awe, determination, and triumph were rated more socially engaging in Hong Kong than in Netherlands.

In terms of the societal evaluations, Hong Kong Chinese participants evaluated the positive emotions to be more appropriate than did the Dutch participants. Specifically, admiration, awe, euphoria, excitement, hope, moved, tender, and triumph were all rated as more *appropriate* in Hong Kong than in Netherlands. In contrast, Dutch participants’ ratings on the extent to which emotions were valued and approved of were generally higher than those of Hong Kong Chinese participants. For evaluation of emotions being *valued* in the society, amusement, gratitude, inspiration, interested, surprise, relief, respect, compassion, peaceful, and connected were all rated as more valued in Netherlands than in Hong Kong, yet awe and triumph were more valued in Hong Kong than in Netherlands. For evaluation of emotions being *approved of* in the society, 10 emotions were rated significantly higher in Netherlands than in Hong Kong: amusement, gratitude, inspiration, interested, relief, respected, contentment, compassion, peaceful, and connected; three emotions were rated as being more approved of in Hong Kong than in Netherlands: awe, moved, and triumph. These findings, thus, yielded partial support for H1, which predicted cultural differences for all of the rating scales except for emotional arousal.

*H2:* We then tested the second hypothesis (H2) that individual perceptions (positivity, arousal, and social engagement) are positively related to evaluations of positive emotions (as appropriate, valued, and approved of) in both Westerners and East Asians. For the Hong Kong Chinese and Dutch samples *separately*, we ran three multilevel models with crossed random effects using the R package lme4 (Bates et al., 2015). Multilevel analyses were conducted because, in H2, we were interested in the link between individual perception and societal evaluation across all of the emotions. Level 1 variables were the six questions participants answered (arousal, positivity, etc.). Level 2 variables were participants and emotions: given that each emotion was rated by all participants, and all participants rated all emotions, there are crossed random effects between emotions and participants. We sought to test the role of perceived positivity, arousal, and social engagement on the extent to which emotions were seen as appropriate (model 1), valued (model 2), and approved of (model 3) in each country. In each model, the outcome was the evaluation of each emotion (appropriate, valued, or approved of), fixed-effect predictors were participants’ perceptions of each emotion (positivity, arousal, and social engagement); emotions and participant ID were entered as crossed random effects. Tables 1A,B shows the results of the multilevel models. The results suggest that, in both Hong Kong and Netherlands, positivity, arousal, and social engagement were all positively related to societal evaluations of emotions, thus supporting H2. In order to compare the different predictors’ effects on the outcome, we used Wald tests on their regression coefficients using the multcomp R package (Hothorn et al., 2008). The relative contributions of positivity, arousal, and social engagement differed across the models. For Dutch participants, across all three evaluations (appropriate, valued, and approved of), the importance of perceived positivity was greater than whether the emotion was perceived as socially engaging, which, in turn, was more influential than whether it was perceived aroused. For participants in Hong Kong, results were more variable across ratings judgments: For predicting whether an emotion was approved, the contributions of judgments of positivity, arousal, and social engagement did not differ from each other. When predicting whether an emotion was seen as appropriate, positivity mattered more than both arousal and degree of social engagement (the latter two did not differ). In predicting whether an emotion was valued, perceived arousal and the extent to which an emotion was perceived as socially engaging mattered more than perceived positivity (arousal and social engagement did not differ). Detailed results can be found in the Supplementary Table 6.

**TABLE 1:** Multilevel regression models of associations between individual evaluations and societal evaluations **(A,B)**, and cultural differences between Hong Kong and Netherlands in the emotion perception–evaluation link of 23 positive emotions **(C)**.

		**Positivity**		**Arousal**		**Social engagement**	
		***b***	***t***	***b***	***t***	***b***	***t***
**(A) Hong Kong**							
Model 1	Appropriate	0.275	22.431***	0.17	14.228***	0.174	13.654***
Model 2	Valued	0.129	8.682***	0.251	17.319***	0.251	16.196***
Model 3	Approved of	0.240	18.586***	0.212	16.872***	0.248	18.421***

		**Positivity**		**Arousal**		**Social engagement**	
		***b***	***t***	***b***	***t***	***b***	***t***

**(B) Netherlands**							
Model 1	Appropriate	0.279	19.962***	0.072	6.857***	0.163	13.857***
Model 2	Valued	0.387	28.626***	0.053	5.221***	0.183	15.980***
Model 3	Approved of	0.396	31.315***	0.036	3.826***	0.200	18.678***

		**Positivity**		**Arousal**		**Social engagement**		**Country^*a*^**		**Positivity × country**		**Arousal × country**		**Social engagement × country**	
		***b***	***t***	***b***	***t***	***b***	***t***	***b***	***t***	***b***	***t***	***b***	***t***	***b***	***t***

Model 1	Appropriate	0.276	22.858***	0.170	14.416 ***	0.174	13.748 ***	−0.12	−3.284**	0.011	0.595	−0.098	−6.318***	−0.012	−0.729
Model 2	Valued	0.122	9.212***	0.259	20.035***	0.250	17.983***	0.165	4.798***	0.280	14.104***	−0.215	−12.665***	−0.056	−3.021**
Model 3	Approved of	0.235	19.806***	0.218	18.775***	0.246	19.741***	0.083	2.814**	0.179	10.067***	−0.189	−12.451***	−0.040	−2.424*

^*a*^Hong Kong as reference, comparing Netherlands vs. Hong Kong. *p < 0.05; **p < 0.01; ***p < 0.001.

**FIGURE 1:**
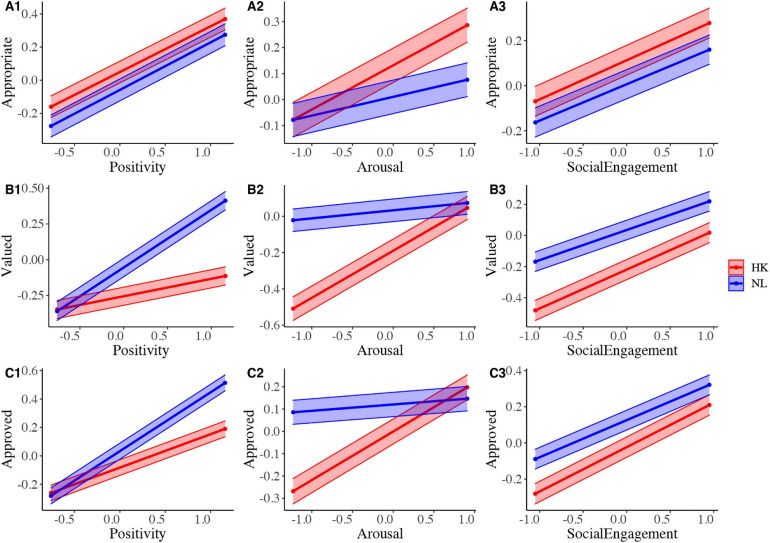
Moderating effect of culture on the associations between individual perceptions and societal evaluations, with 95% confidence intervals displayed. **(A1–A3)** display results for “Appropriate”, **(B1–B3)** display results for “Valued”, and **(C1–C3)** display results for “Approved of”.

*H3:* In the final step, we tested our third hypothesis that there would be cultural differences in the links between individual perceptions and group-level evaluations: positivity (H3a) and arousal (H3b) would be more positively related to societal evaluation in Netherlands than in Hong Kong, and social engagement more positively related to societal evaluation in Hong Kong than in Netherlands (H3c). We added country as a moderator^3^ to the multilevel models conducted in Step 3. The results are presented in Table 1C and visualized in Figure 1. The results suggest that the link between individual evaluations of positivity and the extent to which emotions were valued and approved of was stronger in Netherlands than in Hong Kong (supporting H3a; positivity x country for Model 2 and Model 3 in Table 1C and Figures 1B1,C1). In contrast and contrary to prediction, the link between perceived arousal of a given emotion and whether it was seen as appropriate, valued, and approved of were stronger in Hong Kong than in Netherlands (contrary to H3b; arousal × country interaction for Models 1–3 in Table 1C and Figures 1A2–C2). Finally, the link between whether an emotion was considered to be socially engaging and whether it was valued and approved of was stronger in Hong Kong than in Netherlands (supporting H3c; social engagement × country for Model 2 and Model 3 in Table 1C and Figures 1B3,C3). Overall, our third hypothesis that there would be cultural differences in the links between individual perceptions and group-level evaluations was, thus, partially supported.

### Exploratory Analysis

For exploratory purposes, we tested whether there would be gender differences in participants’ judgments of emotions in each country. Among Hong Kong Chinese, we found no gender difference for any of the six questions. In the Dutch sample, there were significant differences in how men and women judged the extent to which emotions were positive, appropriate, valued, and approved of in all cases with women giving higher ratings (i.e., women tended to think that the positive emotions were more positive, more appropriate, more valued, and more approved of in Dutch society). Detailed statistical analysis and results can be found in the Supplementary Table 8.

## Discussion

This study aimed to test individual perceptions and societal evaluations of distinct positive emotions as well as the role of culture in these processes. Hong Kong Chinese and Dutch students rated 23 positive emotions in terms of how they perceived their positivity, arousal, and social engagement. Participants also answered questions on how they thought each of these emotions was regarded in their societies, that is, whether they were seen as appropriate, valued, and approved of.

We sought to test three hypotheses. First, we hypothesized that there would be cultural similarities in perceptions of arousal and cultural differences in positivity and social engagement perceptions as well as societal evaluations of emotions. However, we observed considerable cross-cultural differences on all six measures between Hong Kong Chinese and the Dutch. Second, we predicted and found that the perceived positivity, arousal, and social engagement of emotions all positively contributed to how emotions were evaluated in both cultures. Third, we expected that there would be cultural differences in the perception–evaluation links. Our third hypothesis received partial support in that we did find that the link between individual perceptions and societal evaluations was moderated by culture. As predicted, greater perceived positivity was associated with more valued and approved emotions in Netherlands than in Hong Kong in line with H3a. In addition, there was a stronger association between the degree of perceived social engagement and societal evaluations of emotions (being valued and approved of) in Hong Kong than in Netherlands, supporting H3c. However, the arousal level of emotions had a stronger positive relationship to positive societal evaluations in the Hong Kong Chinese sample than in the Dutch sample, contradicting H3b.

Our study goes beyond previous research in several ways. First, we tested a large number of distinct positive emotions. Even though some early work collected arousal ratings for a handful of positive emotions in different cultures (Russell et al., 1989), how positive, aroused, and socially engaging/disengaging emotions are is typically assumed rather than empirically established. We hope that the data yielded in this study can offer a helpful empirical reference point for future work on positive emotions.

It is worth noting that we found considerable cultural differences in how positive emotions were judged even when judgments were corrected for cultural differences in scale use. Cultural differences were found for a subset of positive emotions for all judgment types to varying degrees: positivity (10 emotions), arousal (five emotions), social engagement (six emotions), and whether emotions were seen as appropriate (eight emotions), valued (12 emotions), and approved of (13 emotions). Culture, thus, affects evaluations of positive emotions; further work is needed to examine which cultural features and processes drive these effects.

The present study is the first to attempt to directly test relationships between individual judgments and societal evaluations. We found that all three types of individual perceptions (arousal, positivity, engagement) were positively and separately related to societal evaluations. Moreover, we identified cultural differences in these relationships. Specifically, we found that societal evaluations in an individualistic society, such as Netherlands, were driven more by the extent to which positive emotions were seen as more intensely positive, which played a smaller role in a more collectivistic society, such as Hong Kong. This finding is consistent with the more explicit pursuit of positive emotional experiences in individualistic cultures as compared with collectivistic cultures (Eid and Diener, 2001). In Hong Kong, in contrast, we found that societal evaluations were driven more by the extent to which positive emotions were seen as socially engaging. This result is in line with findings in cultural psychology showing that pursuing social harmony is central in collectivistic cultures (Hook et al., 2009). However, we did not find support for the prediction that participants in Netherlands would evaluate high-arousal emotions more and those in Hong Kong would evaluate low-arousal positive emotions more. Instead, we found that, in Hong Kong, emotions that were perceived as more highly aroused were seen as more highly regarded. It is possible that younger individuals in Hong Kong have developed new and different evaluation criteria for positive emotions as compared with their older peers and that our test captured this shift. Another possibility is that this result reflects the adoption of new measures compared with past research on arousal and social evaluations of positive emotions. In the research on ideal affect, participants were asked to judge how often they would ideally like to feel a variety of positive emotional states (Tsai, 2007). Tsai’s work found that culture influenced participants’ ideal affect and related this to the presumed arousal levels of the emotions. In the present study, we explicitly asked participants to rate the arousal level of each emotion and estimated how they thought their society evaluated each of these positive emotions. It would be interesting in future work to directly probe the relationship between individual perceptions of arousal and ideal affect.

It is worth noting several limitations of the present work. First, we did not collect measures of cultural variables, such as self-construal, and, therefore, cannot link the cultural differences that we found to underlying processes. Second, past cultural psychology literature using an intersubjective approach has distinguished the cultural, intersubjective, and individual levels. In the current study, we operationalized country of residence as culture and were, thus, limited to examining culture at the level of country. Moreover, we did not include measures that were independent of our research question, and therefore, corrections for cultural biases were done using the measures used in the analysis. We hope that future research will build on this work in ways that bypass these limitations.

## Conclusion

The present research sought to contribute to understanding of how people from different cultures perceive and evaluate a large variety of positive emotions. We found that, although perceptions and evaluations of emotions vary across cultures, the perceived positivity, arousal, and social engagement contribute to positive evaluations of emotions across cultures. We also showed that perceived positivity matters more for Westerners’ societal evaluations, and arousal and social engagement matter more for East Asians.

## Data Availability Statement

The datasets presented in this study can be found in online repositories. The names of the repository/repositories and accession number(s) can be found below: The data that support the findings of this study are openly available in OSF at https://bit.ly/2ULRIyW.

## Ethics Statement

The studies involving human participants were reviewed and approved by the Department of Psychology, University of Amsterdam. The patients/participants provided their written informed consent to participate in this study.

## Author Contributions

RS: research idea, data collection, data analysis, and manuscript preparation. WH: data collection and manuscript preparation. BH: material translation and manuscript preparation. NS: data collection. TE: material translation. DS: research idea, project supervision, funding acquisition, and manuscript preparation. All authors contributed to the article and approved the submitted version.

## Conflict of Interest

The authors declare that the research was conducted in the absence of any commercial or financial relationships that could be construed as a potential conflict of interest.

## Footnotes

1 Hong Kong is officially called the Hong Kong Special Administrative Region of the People’s Republic of China. In the present work, we use “country” to refer to Hong Kong and Netherlands, respectively, for simplicity.

2 Note that among the six questions, only the socially engaging/disengaging question used a bipolar scale. Given the definition of socially engaging/disengaging, the emotions in our set could be either bringing people together or pushing them further apart. We wanted to capture both situations, so we used a bipolar scale.

3 It is worth noting that country could not be added into the model as level 3 because there are only two countries (Netherlands and Hong Kong). Participants’ country of residence, just as participants’ gender, is an attribute of the participant. It is, therefore, a level 2 variable, not a level 3 variable.
