# Supplementary material for: Perception and Evaluation of 23 Positive Emotions in Hong Kong and the Netherlands
Source: Front Psychol. 2021 May 28;12:579474. doi: 10.3389/fpsyg.2021.579474 (PMC8195569; doi:10.3389/fpsyg.2021.579474)
Supplement: Supplementary file 1 [file Table_1.DOCX]

**Supplementary materials**

Table S1

*23 Positive Emotions With Definitions in English, Dutch and Cantonese*

| Admiration | The feeling when you look up to someone who has excellent abilities or has accomplished impressive things. You have the urge to also achieve such things and be more like this person. | Bewondering | Het gevoel wanneer je opkijkt naar iemand die over uitstekende capaciteiten beschikt of indrukwekkende dingen heeft bereikt. Je hebt de drang om ook zulke dingen te bereiken en meer op deze persoon te lijken. | 欽佩 | 當你仰望一個有出色能力或已經取得令人印象深刻成就的人時，你感到欽佩。 你有衝動也要實現這樣的事情，變得更像這個人。 |
| --- | --- | --- | --- | --- | --- |
| Amusement | The feeling when you encounter something silly, ironic, witty, or absurd, which makes you laugh. You have the urge to be playful and share the joke with others. | Geamuseerd | Het gevoel wanneer je iets stoms, ironisch, grappig of absurd tegenkomt, dat je aan het lachen maakt. Je hebt de neiging om speels te zijn en de grap met anderen te delen. | 感到好笑 | 當你遇到諷刺、滑稽、搞笑的人或事時，你感到好笑；你有想玩的衝動，並且想和別人分享這些笑話。 |
| Awe | The feeling when you encounter something that is greater or more powerful than yourself. You feel overwhelmed and need a moment to adjust. | Ontzag | Het gevoel wanneer je iets tegenkomt dat groter of sterker is dan jezelf. Je voelt je overweldigd en hebt een moment nodig om je aan te passen. | 敬畏 | 當你遇到比你更強大或更強大的東西時，你感到敬畏。 你感到不知所措，需要一點時間來調整。 |
| Determination | The feeling when you are working on a demanding problem or task that you believe you can handle. You feel stimulated to accomplish it. | Vastberaden | Het gevoel wanneer u werkt aan een veeleisend probleem of taak waarvan u denkt dat u het aankan. U voelt zich gestimuleerd om het te bereiken | 堅定 | 當你正在處理一個你認爲可以處理的苛刻問題或任務時，你感到堅定。 你覺得有動力完成它。 |
| Euphoria | The feeling when something very good just happened to you. You have the urge to celebrate and share it with others. | Euforie | Het gevoel wanneer iets heel goeds met je is gebeurd. Je hebt de drang om het te vieren en het met anderen te delen. | 興高采烈 | 一件好事發生在你身上，你感覺興高采烈。 你有衝動來慶祝並與他人分享。 |
| Excitement | The feeling when you expect something good or nice will happen to you. You cannot stop thinking about this. | Opwinding | Het gevoel wanneer je verwacht dat iets goeds of leuks met jou zal gebeuren. Je kunt niet stoppen met hieraan denken. | 興奮 | 興奮是當你期待一些好事發生在你身上的激動感覺。你控制不住一直想這些事。 |
| Gratitude | The feeling when you think that someone has gone out of their way to do something good or nice for you. You have the urge to do something back and get closer to this person. | Dankbaarheid | Het gevoel wanneer je denkt dat iemand zijn uiterste best heeft gedaan om iets goeds of leuks voor je te doen. Je hebt de drang om iets terug te doen. | 感激 | 感激指因爲別人的好意或幫助而對其有好感；對於施恩者懷有熱烈友好的感情, 促使人去接近這個人以報答恩情 |
| Hope | The feeling when you believe that a desired event has a possibility of happening. You keep thinking about how good it would be if the event actually happened. | Hoop | Het gevoel wanneer u gelooft dat een gewenste gebeurtenis de mogelijkheid heeft om te gebeuren. Je blijft maar denken aan hoe goed het zou zijn als de gebeurtenis echt zou plaatsvinden. | 希望 | 當你相信所期望的事件有可能發生時, 你感覺到有希望。 你會一直在想如果事件真的發生會有多好。 |
| Inspiration | The feeling when you suddenly get a new idea or insight, or see the world in a different light. You have the urge to express or actualize this new insight. | Inspiratie | Het gevoel wanneer je plots een nieuw idee of inzicht krijgt, of de wereld in een ander licht ziet. Je hebt de drang dit nieuwe inzicht uit te drukken of te actualiseren. | 靈感 | 當你有靈感時，你突然得到新的想法或見解，或突然以不同的方式看待世界時。 你有衝動來表達或實現這種新的見解。 |
| Interested | The feeling when you encounter something new and relevant that you do not immediately understand. You have the urge to find out more about it. | Interesse | Het gevoel wanneer je iets nieuws en relevants tegenkomt dat je niet meteen begrijpt. Je hebt de drang er meer over te weten te komen. | 感興趣的 | 當你遇到一些你不明白的但和你相關的新事物或人時,你對它/他/她感興趣.你有衝動想知道更多關於它/他/她的信息。 |
| Moved | The feeling when you encounter something very beautiful, meaningful, or bittersweet. Tears well up in your eyes and you feel overcome with warm feelings. | Ontroering | Het gevoel wanneer je iets heel moois, betekenisvol of bitterzoet tegenkomt. Tranen vullen je ogen en je voelt je overweldigd door warme gevoelens. | 感動 | 當你遇到一些非常美麗，有意義或苦樂參半事情時，你受到感動。 在你眼中可能會充滿淚水，你會感受到溫暖。 |
| Positive surprise | The feeling when you realize something good or nice just happened, which you did not expect. You need a moment to take in the good news. | Positieve verassing | Het gevoel wanneer je je realiseert dat er iets goeds of leuks gebeurd wat je niet had verwacht. Je hebt een moment nodig om het goede nieuws op te nemen. | 驚喜 | 當你沒有預料到的好事發生時，你感到驚喜；你可能需要一點時間消化這個好消息 |
| Pride | The feeling when you (or someone close to you) possess or have accomplished something that other people find praiseworthy. | Trots | Het gevoel wanneer u (of een naaste van u) iets bezit of heeft bereikt dat andere mensen lovenswaardig vinden. | 自豪 | 當你（或和你親近的某個人）擁有或成就了其他人認爲值得贊美的事情時，你感覺自豪 |
| Relief | The feeling when an unpleasant experience is finally over, or when you find out that something you had dreaded has not happened (or will not happen). You can finally take your mind off it. | Opluchting | Het gevoel wanneer een onaangename ervaring ten einde is, of wanneer je erachter komt dat iets waar je bang voor was niet is gebeurd (of niet zal gebeuren). Je kunt je gedachten er eindelijk van af wenden. | 解脫 | 當不愉快的經歷終於結束，或者當你發現你曾經感到恐懼的事情還沒有發生（或不會發生）時，你會感覺解脫。 你終於可以放下心思了。 |
| Respected | The feeling when you're valued and recognised, treated politely, and deemed worthy by others confirmed through listening, hearing, and understanding, even with disagreements. | Gerespecteerd | Het gevoel wanneer je wordt gewaardeerd en herkend, beleefd wordt behandeld en waardig wordt bevonden door anderen, bevestigd door luisteren, horen en begrijpen, zelfs met meningsverschillen. | 尊重 | 當你受到重視和認可，被有禮貌地對待並被其他人視爲有價值時，你感覺受到尊重。當別人尊重你的時候，即使他們持不同意見，他們也會努力傾聽和理解你。 |
| Schadenfreude | The feeling when something bad happens to another person. You enjoy this because you dislike the person, because you think the person deserves it, or because it is somehow good for you. | Leedvermaak | Het gevoel wanneer er iets ergs met een ander gebeurt. Je geniet ervan omdat je de persoon niet leuk vindt, omdat je denkt dat de persoon het verdient, of omdat het op een of andere manier goed voor je is. | 幸災樂禍 | 幸災樂禍指的是在別人遇到災禍時感到高興。你感覺到幸災樂禍因爲你不喜歡這個人，或者認爲這個人活該，或者別人遇到災禍能給你帶來好處。 |
| Sensory pleasure | The feeling when something happens that pleases your senses. | Zintuigelijk Genot | Het gevoel wanneer iets gebeurt dat uw zintuigen behaagd. | 感官愉悅 | 你的感官感受到愉悅 |
| Tenderness | The feeling when you encounter someone or something that seems cute, vulnerable, or childlike in appearance or behavior. You have the urge to nurture and care for this person or thing. | Tederheid | Het gevoel wanneer je iemand of iets tegenkomt dat schattig, kwetsbaar of kinderlijk lijkt qua uiterlijk of gedrag. Je hebt de drang om deze persoon of dat ding te koesteren en te verzorgen. | 溫柔的愛 | 當你遇到某個可愛、看起來脆弱、或者幼小的人或者東西時，你感覺到溫柔的愛；你有照顧這個人或者東西的衝動。 |
| Triumph | The feeling of release and a great joy, after a successful ending of a struggle or contest. | Triomfantelijk | Het gevoel van bevrijding en een grote vreugde, na een succesvol einde van een worsteling of wedstrijd. | 獲勝感 | 當你贏得比賽或者戰勝困難之後，感覺到勝利感；你感覺到了極大的成功、快樂和放鬆 |
| Contentment | The feeling when you are pleased with your situation and not hoping for change or improvement. | Tevredenheid | Het gevoel wanneer u tevreden bent met uw situatie en niet op verandering of verbetering hoopt | 滿足 | 當你對自己的情況感到滿意而不尋求改變或改善時的感覺 |
| Compassion | The feeling that arises in witnessing another's suffering and that motivates a subsequent desire to help. | Compassie | Het gevoel dat ontstaat bij het zien van andermans leed en dat een volgend verlangen om te helpen motiveert. | 同情 | 同情是在目睹另一個人的痛苦時産生的感覺，並且同情激發了隨後的幫助欲望 |
| Peaceful | The feeling of serenity and calmness; worried by nothing. | Vredig | Het gevoel van rust en kalmte; bezorgd door niets. | 安寧 | 寧靜平靜的感覺; 沒有煩心事 |
| Connected | Feeling connected to people who care about us. | verbonden voelen | Zich verbonden voelen met mensen die om ons geven. | 有聯繫的 | 感覺與關心我們的人有著聯繫。 |

Table S2

*Hong Kong Chinese (HK) and Dutch (NL) participants’ Ratings for 23 Positive Emotions on six 9-Point Likert Scales (Raw Scores)**

|  | |  | **Positivity** | **Arousal** | **Social Engagement** | **Appropriate** | **Valued** | **Approved of** |
| --- | --- | --- | --- | --- | --- | --- | --- | --- |
| **Admiration** | HK | | 6.93 (1.56) | 6.16 (1.60) | 6.55 (1.47) | 6.89 (1.42) | 6.11 (1.72) | 6.57 (1.49) |
|  | NL | | 6.87 (1.66) | 6.08 (1.87) | 6.46 (1.84) | 6.60 (1.75) | 6.75 (1.65) | 6.80 (1.70) |
| **Amusement** | HK | | 6.23 (1.79) | 6.53 (1.59) | 6.53 (1.56) | 6.47 (1.52) | 6.2 (1.64) | 6.38 (1.55) |
|  | NL | | 7.40 (1.62) | 6.59 (1.85) | 7.30 (1.55) | 6.99 (1.59) | 7.06 (1.59) | 7.26 (1.52) |
| **Awe** | HK | | 5.62 (1.78) | 5.86 (1.62) | 5.95 (1.51) | 6.02 (1.55) | 5.64 (1.76) | 5.93 (1.67) |
|  | NL | | 4.91 (2.08) | 5.10 (2.16) | 4.3 (2.06) | 5.47 (1.85) | 5.03 (1.85) | 5.39 (1.82) |
| **Determination** | HK | | 7.07 (1.54) | 6.14 (1.68) | 6.31 (1.54) | 6.84 (1.56) | 6.41 (1.60) | 6.66 (1.50) |
|  | NL | | 7.15 (1.58) | 6.21 (1.91) | 5.72 (1.87) | 6.95 (1.67) | 7.04 (1.67) | 7.15 (1.60) |
| **Euphoria** | HK | | 7.40 (1.34) | 6.92 (1.48) | 6.8 (1.46) | 6.99 (1.49) | 6.44 (1.54) | 6.74 (1.51) |
|  | NL | | 7.40 (1.61) | 7.21 (1.72) | 6.93 (1.79) | 6.77 (1.67) | 6.74 (1.67) | 6.93 (1.57) |
| **Excitement** | HK | | 7.07 (1.39) | 6.92 (1.53) | 6.56 (1.31) | 6.71 (1.49) | 6.29 (1.61) | 6.62 (1.50) |
|  | NL | | 7.01 (1.66) | 7.28 (1.69) | 6.39 (1.65) | 6.51 (1.6) | 6.51 (1.61) | 6.60 (1.58) |
| **Gratitude** | HK | | 7.56 (1.41) | 6.68 (1.48) | 7.07 (1.40) | 7.18 (1.52) | 6.41 (1.75) | 6.94 (1.54) |
|  | NL | | 7.76 (1.53) | 6.28 (2.22) | 7.73 (1.58) | 7.39 (1.66) | 7.57 (1.66) | 7.71 (1.59) |
| **Hope** | HK | | 7.43 (1.59) | 6.38 (1.8) | 6.68 (1.55) | 6.84 (1.83) | 6.21 (1.91) | 6.50 (1.73) |
|  | NL | | 7.02 (1.76) | 6.18 (1.96) | 6.52 (1.73) | 6.53 (1.68) | 6.68 (1.62) | 6.91 (1.65) |
| **Inspiration** | HK | | 6.72 (1.41) | 6.01 (1.73) | 5.81 (1.69) | 6.53 (1.61) | 5.54 (1.94) | 5.95 (1.75) |
|  | NL | | 7.54 (1.59) | 6.66 (1.94) | 6.36 (1.71) | 6.94 (1.68) | 7.07 (1.52) | 7.13 (1.63) |
| **Interested** | HK | | 6.83 (1.29) | 6.39 (1.45) | 6.49 (1.36) | 6.76 (1.33) | 6.26 (1.53) | 6.57 (1.42) |
|  | NL | | 7.59 (1.49) | 6.32 (1.98) | 7.12 (1.72) | 7.31 (1.52) | 7.35 (1.53) | 7.46 (1.55) |
| **Moved** | HK | | 7.17 (1.48) | 6.82 (1.65) | 6.88 (1.45) | 6.93 (1.46) | 6.43 (1.70) | 6.85 (1.52) |
|  | NL | | 6.82 (1.63) | 6.38 (1.9) | 6.75 (1.71) | 6.46 (1.67) | 6.49 (1.62) | 6.67 (1.62) |
| **Positive surprise** | HK | | 6.64 (1.37) | 6.73 (1.48) | 6.48 (1.31) | 6.64 (1.38) | 6.20 (1.64) | 6.68 (1.38) |
|  | NL | | 7.35 (1.51) | 7.02 (1.68) | 6.77 (1.62) | 6.82 (1.58) | 6.98 (1.57) | 7.20 (1.53) |
| **Pride** | HK | | 6.63 (1.53) | 6.32 (1.52) | 6.16 (1.51) | 6.57 (1.43) | 6.17 (1.6) | 6.43 (1.49) |
|  | NL | | 6.86 (1.71) | 6.50 (1.90) | 5.95 (2.12) | 6.67 (1.66) | 6.43 (1.83) | 6.57 (1.78) |
| **Relief** | HK | | 6.00 (1.93) | 6.17 (1.76) | 5.72 (1.79) | 6.12 (1.71) | 5.77 (1.91) | 5.90 (1.81) |
|  | NL | | 6.81 (1.69) | 6.21 (2.10) | 6.09 (1.67) | 6.6 (1.51) | 6.63 (1.52) | 6.77 (1.54) |
| **Respected** | HK | | 7.45 (1.54) | 6.36 (1.76) | 7.04 (1.64) | 7.21 (1.73) | 6.47 (1.94) | 6.81 (1.69) |
|  | NL | | 7.48 (1.68) | 6.26 (2.21) | 7.21 (1.81) | 7.23 (1.63) | 7.42 (1.59) | 7.55 (1.54) |
| **Schadenfreude** | HK | | 2.89 (1.99) | 4.93 (2.06) | 4.41 (2.35) | 4.07 (2.08) | 4.67 (1.98) | 4.15 (2.20) |
|  | NL | | 3.18 (2.22) | 4.57 (2.34) | 3.30 (2.32) | 4.26 (2.27) | 3.72 (2.13) | 3.82 (2.20) |
| **Sensory pleasure** | HK | | 6.51 (1.51) | 6.3 (1.71) | 6.07 (1.61) | 6.46 (1.55) | 5.91 (1.86) | 6.17 (1.70) |
|  | NL | | 6.82 (1.73) | 6.46 (1.83) | 6.28 (1.7) | 6.34 (1.67) | 6.32 (1.73) | 6.35 (1.75) |
| **Tenderness** | HK | | 7.20 (1.50) | 6.35 (1.73) | 6.73 (1.47) | 6.85 (1.57) | 6.13 (1.83) | 6.54 (1.61) |
|  | NL | | 6.85 (1.6) | 5.77 (2.04) | 7.06 (1.63) | 6.34 (1.73) | 6.47 (1.65) | 6.68 (1.63) |
| **Triumph** | HK | | 6.79 (1.51) | 6.87 (1.52) | 6.52 (1.47) | 7.01 (1.45) | 6.73 (1.65) | 6.86 (1.49) |
|  | NL | | 6.63 (1.75) | 6.55 (1.77) | 5.74 (1.94) | 6.47 (1.76) | 6.27 (1.69) | 6.46 (1.76) |
| **Contentment** | HK | | 6.85 (1.7) | 6.14 (1.79) | 6.37 (1.63) | 6.62 (1.74) | 6.33 (1.74) | 6.44 (1.75) |
|  | NL | | 7.36 (1.59) | 5.89 (2.21) | 6.53 (1.65) | 6.74 (1.61) | 6.90 (1.64) | 7.13 (1.54) |
| **Compassion** | HK | | 6.44 (1.7) | 6.14 (1.62) | 6.61 (1.46) | 6.66 (1.52) | 5.97 (1.68) | 6.25 (1.58) |
|  | NL | | 7.32 (1.75) | 6.03 (1.99) | 7.58 (1.72) | 6.82 (1.85) | 6.96 (1.85) | 7.21 (1.71) |
| **Peaceful** | HK | | 6.85 (1.53) | 5.29 (2.09) | 5.7 (1.88) | 6.33 (1.94) | 5.57 (1.96) | 6.14 (1.69) |
|  | NL | | 7.43 (1.61) | 5.30 (2.52) | 6.31 (1.79) | 6.45 (1.83) | 6.72 (1.68) | 7.02 (1.58) |
| **Connected** | HK | | 7.02 (1.5) | 6.27 (1.66) | 7.07 (1.64) | 6.78 (1.51) | 6.25 (1.81) | 6.55 (1.63) |
|  | NL | | 7.52 (1.61) | 6.47 (1.99) | 7.89 (1.52) | 7.14 (1.72) | 7.27 (1.7) | 7.38 (1.64) |

*** For five out of the six questions (here: columns), options ranged from 1 (not at all) to 9 (very much). For the question on social engagement/disengagement, options ranged from 1 (very disengaging) to 9 (very engaging).

Table S3

*Hong Kong Chinese (HK) and Dutch (NL) Participants’ Mean Ratings for 22 Positive Emotions (Corrected for Cultural Differences in Response Tendencies) ^a^*

|  |  | **positivity** | **arousal** | **social engagement** | **appropriate** | **valued** | **approved of** |
| --- | --- | --- | --- | --- | --- | --- | --- |
| **Admiration** | HK | .26 (.94) | -.21 (.96) | .03 (.88) | .24 (.85) | -.23 (1.03) | .04 (.89) |
|  | NL | .08 (.91) | -.36 (1.02) | -.15 (1.01) | -.07 (.96) | .01 (.90) | .04 (.93) |
| **Amusement** | HK | -.16 (1.08) | .02 (.95) | .02 (.94) | -.02 (.91) | -.18(.99) | -.07 (.94) |
|  | NL | .37 (.88) | -.08 (1.01) | .31 (.85) | .14 (.87) | .18 (.87) | .29 (.83) |
| **Awe** | HK | -.53 (1.07) | -.38 (.97) | -.33 (.91) | -.28 (.93) | -.52 (1.06) | -.34 (1.00) |
|  | NL | -1.00 (1.14) | -.89 (1.18) | -1.33 (1.13) | -.69 (1.01) | -.93 (1.01) | -.73 (.99) |
| **Determination** | HK | .34 (.92) | -.21 (1.01) | -.11 (.93) | .21 (.94) | -.05 (.96) | .10 (.90) |
|  | NL | .23 (.86) | -.28 (1.04) | -.55 (1.02) | .12 (.91) | .17 (.91) | .23 (.87) |
| **Euphoria** | HK | .54 (.81) | .26 (.89) | .18 (.88) | .30 (.90) | -.04 (.92) | .15 (.91) |
|  | NL | .36 (.88) | .26 (.94) | .11 (.98) | .02 (.91) | .01 (.91) | .11 (.86) |
| **Excitement** | HK | .34 (.84) | .25 (.92) | .04 (.79) | .13 (.89) | -.12 (.97) | .08 (.90) |
|  | NL | .15 (.90) | .30 (.92) | -.18 (.90) | -.12 (.87) | -.12 (.88) | -.07 (.86) |
| **Gratitude** | HK | .64 (.85) | .11 (.89) | .34 (.84) | .41 (.91) | -.05 (1.05) | .27 (.93) |
|  | NL | .56 (.84) | -.24 (1.21) | .55 (.86) | .36 (.90) | .46 (.91) | .54 (.87) |
| **Hope** | HK | .56 (.96) | -.07 (1.09) | .11 (.93) | .21 (1.10) | -.17 (1.15) | .00 (1.04) |
|  | NL | .16 (.96) | -.30 (1.07) | -.11 (.94) | -.11 (.92) | -.03 (.89) | .10 (.90) |
| **Inspiration** | HK | .13 (.85) | -.29 (1.04) | -.41 (1.02) | .02 (.97) | -.58 (1.16) | -.33 (1.05) |
|  | NL | .44 (.87) | -.04 (1.06) | -.20 (.93) | .12 (.92) | .19 (.83) | .22 (.89) |
| **Interested** | HK | .20 (.78) | -.07 (.87) | .00 (.82) | .16 (.80) | -.14 (.92) | .04 (.86) |
|  | NL | .47 (.81) | -.22 (1.08) | .21 (.94) | .32 (.83) | .34 (.84) | .40 (.85) |
| **Moved** | HK | .40 (.89) | .19 (.99) | .23 (.87) | .26 (.88) | -.04 (1.02) | .21 (.91) |
|  | NL | .05 (.89) | -.19 (1.04) | .01 (.93) | -.15 (.91) | -.13 (.89) | -.03 (.88) |
| **Positive surprise** | HK | .09 (.82) | .14 (.89) | -.01 (.79) | .09 (.83) | -.18 (.99) | .11 (.83) |
|  | NL | .34 (.83) | .16 (.92) | .02 (.88) | .05 (.86) | .14 (.86) | .26 (.84) |
| **Pride** | HK | .08 (.92) | -.11 (.91) | -.20 (.91) | .04 (.86) | -.20 (.96) | -.04 (.90) |
|  | NL | .07 (.93) | -.12 (1.03) | -.42 (1.15) | -.03 (.91) | -.16 (1.00) | -.09 (.97) |
| **Relief** | HK | -.30 (1.16) | -.20 (1.06) | -.47 (1.08) | -.22 (1.03) | -.44 (1.15) | -.36 (1.09) |
|  | NL | .04 (.92) | -.29 (1.15) | -.35 (.91) | -.07 (.82) | -.05 (.83) | .02 (.84) |
| **Respected** | HK | .58 (.93) | -.08 (1.06) | .33 (.99) | .43 (1.04) | -.02 (1.17) | .19 (1.02) |
|  | NL | .41 (.92) | -.25 (1.2) | .26 (.99) | .28 (.89) | .37 (.87) | .45 (.84) |
| **Sensory pleasure** | HK | .01 (.91) | -.12 (1.03) | -.26 (.97) | -.02 (.93) | -.36 (1.12) | -.20 (1.03) |
|  | NL | .05 (.95) | -.15 (1.00) | -.24 (.93) | -.21 (.91) | -.22 (.94) | -.21 (.96) |
| **Tenderness** | HK | .42 (.90) | -.09 (1.04) | .14 (.88) | .21 (.94) | -.22 (1.10) | .03 (.97) |
|  | NL | .07 (.88) | -.52 (1.11) | .18 (.89) | -.21 (.94) | -.14 (.90) | -.02 (.89) |
| **Triumph** | HK | .17 (.91) | .23 (.91) | .02 (.89) | .31 (.87) | .14 (.99) | .22 (.90) |
|  | NL | -.05 (.95) | -.10 (.97) | -.54 (1.06) | -.14 (.96) | -.25 (.92) | -.15 (.96) |
| **Contentment** | HK | .21 (1.02) | -.21 (1.08) | -.07 (.98) | .07 (1.05) | -.10 (1.05) | -.03 (1.05) |
|  | NL | .34 (.87) | -.46 (1.21) | -.11 (.9) | .00 (.88) | .09 (.90) | .22 (.84) |
| **Compassion** | HK | -.04 (1.02) | -.22 (.98) | .07 (.88) | .10 (.92) | -.32 (1.01) | -.15 (.95) |
|  | NL | .32 (.95) | -.38 (1.09) | .47 (.94) | .05 (1.01) | .13 (1.01) | .26 (.94) |
| **Peaceful** | HK | .21 (.92) | -.73 (1.26) | -.48 (1.13) | -.10 (1.16) | -.56 (1.18) | -.21 (1.02) |
|  | NL | .38 (.88) | -.78 (1.38) | -.23 (.98) | -.15 (1.00) | .00 (.92) | .16 (.86) |
| **Connected** | HK | .32 (.90) | -.14 (1.00) | .34 (.99) | .17 (.91) | -.15 (1.09) | .03 (.98) |
|  | NL | .43 (.88) | -.14 (1.09) | .63 (.83) | .22 (.94) | .29 (.93) | .35 (.90) |

1. For each country, we first subtracted the grand mean (the mean across all items and all individuals in the group) from each response, and then divided it by the grand standard deviation (the standard deviation across all items and individuals in that group). Therefore, 0 denotes the mean score of all six ratings in that country; a response above 0 thus does not mean it is above this specific question’s mean score.

Table S4^[[1]](#footnote-2)^
*A. Cronbach’s alpha for All Six Questions*

*B. Average Ratings for All Six Questions After Correcting for Cultural Response Biases*

| **A** | positivity | arousal | social engagement | appropriate | valued | approved of |
| --- | --- | --- | --- | --- | --- | --- |
| Hong Kong | .90 | .91 | .89 | .93 | .91 | .90 |
| The Netherlands | .92 | .91 | .86 | .94 | .92 | .93 |
|  |  |  |  |  |  |  |
| **B** | positivity | arousal | social engagement | appropriate | valued | approved of |
| Hong Kong | .204 | -.087 | -.023 | .123 | -.205 | -.012 |
| The Netherlands | .195 | -.23 | -.075 | -.012 | .016 | .107 |

Table S5

*Results of Tests of Cultural differences on emotion ratings (Hypothesis 1).* Multivariate effect of culture on emotion ratings (A) and Bonferroni-Holm Corrected p Values for Cultural Comparisons of Hong Kong Chinese versus Dutch participants of Ratings on 22 Positive Emotions (B).

| A | positivity | arousal | social engagement | appropriate | valued | approved of |
| --- | --- | --- | --- | --- | --- | --- |
| F | 12.059 | 5.0292 | 10.992 | 8.359 | 12.607 | 12.059 |
| p value | <.001 | <.001 | <.001 | <.001 | <.001 | <.001 |

| B | positivity | arousal | social engagement | appropriate | valued | approved of |
| --- | --- | --- | --- | --- | --- | --- |
| Admiration | .254 | 1.000 | .306 | **.004** | .051 | 1.000 |
| Amusement | **.000** | 1.000 | **.006** | .563 | **.000** | **.000** |
| Awe | **.000** | **.000** | **.000** | **.000** | **.000** | **.000** |
| Determination | .793 | 1.000 | **.000** | 1.000 | .072 | .732 |
| Euphoria | .193 | 1.000 | 1.000 | **.013** | 1.000 | 1.000 |
| Excitement | .156 | 1.000 | .062 | **.029** | 1.000 | .500 |
| Gratitude | .942 | **.005** | .107 | 1.000 | **.000** | **.011** |
| Hope | **.000** | .291 | .107 | **.010** | .862 | 1.000 |
| Inspiration | **.001** | .116 | .153 | 1.000 | **.000** | **.000** |
| Interested | **.003** | 1.000 | .101 | .395 | **.000** | **.000** |
| Moved | **.000** | **.001** | .107 | **.000** | 1.000 | **.027** |
| Positive surprise | **.009** | 1.000 | 1.000 | 1.000 | **.001** | .437 |
| Pride | 1.000 | 1.000 | .155 | 1.000 | 1.000 | 1.000 |
| Relief | **.005** | 1.000 | 1.000 | .700 | **.000** | **.000** |
| Respected | .303 | 1.000 | 1.000 | .734 | **.000** | **.027** |
| Sensory pleasure | 1.000 | 1.000 | 1.000 | .330 | .882 | 1.000 |
| Tenderness | **.000** | **.000** | 1.000 | **.000** | 1.000 | 1.000 |
| Triumph | .077 | **.003** | **.000** | **.000** | **.000** | **.000** |
| Contentment | .793 | .291 | 1.000 | 1.000 | .235 | **.033** |
| Compassion | **.001** | 1.000 | **.000** | 1.000 | **.000** | **.000** |
| Peaceful | .272 | 1.000 | .107 | 1.000 | **.000** | **.000** |
| Connected | .793 | 1.000 | **.007** | 1.000 | **.000** | **.002** |

Table S6

*Comparison of the Effects of individual perceptions (positivity, arousal and social engagement) on evaluations (appropriateness, valued, approved of) via Wald Tests on the Regression Coefficients for Hong Kong Chinese (A, HK) and Dutch (B, NL) participants*

| \| (A) HK \| Comparison \| Estimate \| Std. Error \| z value \| Pr(>\|z\|) \| \| --- \| --- \| --- \| --- \| --- \| --- \| \| appropriate \| positivity - arousal \| .105 \| .019 \| 5.593 \| 2.23e-08 *** \| \| positivity – social engagement \| .101 \| .020 \| 4.988 \| 6.11e-07 *** \| \| arousal - social engagement \| -.004 \| .020 \| -.215 \| .829 \| \| value \| positivity - arousal \| -.121 \| .023 \| -5.309 \| 1.10e-07 *** \| \| positivity - social engagement \| -.122 \| .025 \| -4.955 \| 7.22e-07 *** \| \| arousal - social engagement \| -.0004 \| .024 \| -.015 \| .988 \| \| approved of \| positivity - arousal \| .028 \| .020 \| 1.429 \| .153 \| \| positivity - social engagement \| -.008 \| .021 \| -.376 \| .707 \| \| arousal - social engagement \| -.036 \| .021 \| -1.708 \| .088. \| \|  \|  \|  \|  \|  \|  \| \| (B) NL \| Comparison \| Estimate \| Std. Error \| z value \| Pr(>\|z\|) \| \| appropriate \| positivity - arousal \| .207 \| .020 \| 10.437 \| < 2e-16*** \| \| positivity - social engagement \| .116 \| .022 \| 5.306 \| 1.12e-07 *** \| \| arousal - social engagement \| -.091 \| .017 \| -5.494 \| 3.92e-08 *** \| \| value \| positivity - arousal \| .334 \| .019 \| 17.372 \| < 2e-16 *** \| \| positivity - social engagement \| .204 \| .021 \| 9.600 \| < 2e-16 *** \| \| arousal - social engagement \| -.130 \| .016 \| -8.058 \| 6.66e-16 *** \| \| approved of \| positivity - arousal \| .360 \| .018 \| 20.006 \| <2e-16 *** \| \| positivity - social engagement \| .197 \| .020 \| 9.889 \| <2e-16 *** \| \| arousal - social engagement \| -.163 \| .015 \| -10.842 \| <2e-16 *** \| |  |  |  |  |  |
| --- | --- | --- | --- | --- | --- | --- | --- | --- | --- | --- | --- | --- | --- | --- | --- | --- | --- | --- | --- | --- | --- | --- | --- | --- | --- | --- | --- | --- | --- | --- | --- | --- | --- | --- | --- | --- | --- | --- | --- | --- | --- | --- | --- | --- | --- | --- | --- | --- | --- | --- | --- | --- | --- | --- | --- | --- | --- | --- | --- | --- | --- | --- | --- | --- | --- | --- | --- | --- | --- | --- | --- | --- | --- | --- | --- | --- | --- | --- | --- | --- | --- | --- | --- | --- | --- | --- | --- | --- | --- | --- | --- | --- | --- | --- | --- | --- | --- | --- | --- | --- | --- | --- | --- | --- | --- | --- | --- | --- | --- | --- | --- | --- | --- | --- | --- | --- | --- | --- | --- |

To measure the amount of multicollinearity in the regression models, we examined collinearity using Variance Inflation Factor (VIF) for the models tested in H2 and H3 using the car package in R (Fox & Weisberg, 2018). Using the standard VIF cut-off score of 5 (O’Brien, 2007), our results suggested that there was no collinearity in the models (see Table S7 for the full VIF results).

Table S7

*VIF Results from Collinearity Tests of Regression Models in Hong Kong Chinese (A), and Dutch (B, NL) participants, and for tests of cultural differences (C).*

1. HK (H2)

|  |  | **positivity** | **arousal** | **social engagement** |
| --- | --- | --- | --- | --- |
| **Model 1** | appropriate | 1.246 | 1.270 | 1.340 |
| **Model 2** | valued | 1.253 | 1.279 | 1.347 |
| **Model 3** | approved of | 1.261 | 1.287 | 1.359 |

1. NL (H2)

|  |  | **positivity** | **arousal** | **social engagement** |
| --- | --- | --- | --- | --- |
| **Model 1** | appropriate | 1.484 | 1.203 | 1.362 |
| **Model 2** | valued | 1.491 | 1.212 | 1.368 |
| **Model 3** | approved of | 1.489 | 1.209 | 1.366 |

1. Cultural differences on the relationship between emotion perception and evaluation (H3)

|  |  | **positivity** | **arousal** | **social engagement** | **country** | **positivity x country** | **arousal x country** | **social engagement x country** |
| --- | --- | --- | --- | --- | --- | --- | --- | --- |
| **Model 1** | appropriate | 2.334 | 2.762 | 2.932 | 1.019 | 2.644 | 2.698 | 3.018 |
| **Model 2** | valued | 2.361 | 2.791 | 2.955 | 1.029 | 2.674 | 2.725 | 3.035 |
| **Model 3** | approved of | 2.368 | 2.798 | 2.961 | 1.031 | 2.681 | 2.731 | 3.040 |

Exploratory analysis: Do judgements of positive emotions differ between genders?

To examine this question, we ran t-tests comparing judgements between genders in each country, with scores reflecting the average corrected scores across the 22 emotions. For example, for every participant, we averaged the positivity scores for 22 emotions to create a composite emotion positivity rating. Then in each country, we ran independent t-test comparing men and women on each of the judgements. Since we conducted 12 independent t-test, we adjusted the p-values by using the Bonferroni-Holm method. Results indicate that in Hong Kong, there were no gender difference for any of the six questions. In the Netherlands, there were gender differences on judgments of positivity, appropriate, valued, and approved of. Specifically, compared to men, women judged positive emotions to be more positive, as well as more appropriate, more valued, and more approved of in Dutch society.

Table S8

Gender Differences on judgement of emotions in Hong Kong (HK) and the Netherlands (NL)

|  |  | **positivity** | | **arousal** | | **social engagement** | | **appropriate** | | **valued** | | **approved of** | |
| --- | --- | --- | --- | --- | --- | --- | --- | --- | --- | --- | --- | --- | --- |
|  |  | mean | t | mean | t | mean | t | mean | t | mean | t | mean | t |
| **HK** | M^a^ (n = 113) | .237 | t (231.54) = .945,  p = .346 | -.058 | t (232.75) = .810,  p = .419 | .024 | t (232.87) = 1.467,  p = .144 | .148 | t(232.71)=.489,  p = .625 | -.173 | t (233) = .772,  p = .441 | .035 | t (232.93) = 1.346,  p = .180 |
|  | W^b^ (n = 122) | .173 |  | -.119 |  | -.073 |  | .110 |  | -.235 |  | -.062 |  |
| **NL** | M (n = 122) | .082 | t (241.89) = -3.151,  p = .002  (adjusted p = .006) | -.289 | t (248.63) = -1.325,  p = .186 | -.118 | t (242.13) = -1.368,  p = .173 | -.148 | t(238.47) = -3.514,  p = .001  (adjusted p = .004) | -.116 | t (242.95) = -3.673,  p <.001  (adjusted p = .006) | -.023 | t (241.38) = -3.502,  p = .001  (adjusted p = .016) |
|  | W (n = 129) | .299 |  | -.182 |  | -.036 |  | .113 |  | .135 |  | .224 |  |

1. M = men
2. W = women
3. Note: In both Hong Kong and the Netherlands, very few participants preferred not to report their gender information, therefore, in the current analysis, we only compared men and women when judging emotions.

1. It is worth noting that after correcting for cultural response biases, the average score for all answers from Hong Kong Chinese/Dutch participants was zero (sum of scores in the Hong Kong Chinese sample equal to zero, same applied to the Dutch sample) [↑](#footnote-ref-2)
